# 

**Published:** 1888-06

**Authors:** 


### INDEX TO VOLUME LVI.

### A

PAGE

Abdomen, Gunshot Wounds of. Murphy
Abdominal Surgery, Two Cases of. Bogue ..... 4
Abstracts and Extracts..28, 116, 177, 232, [illegible]4, 394
Acetanilid. McConnell ..... 22
Age, Old, The Pathology of ..... 56
Air, The Toxic Principle of Expired ..... 321
Alcoholism, Experiments on Hereditary ..... 392
American Anthropologist, The ..... 200
American Medical Association ..... 328
American Medical Association, The ..... 200
American Medical Association, Thirty-ninth Annual Meeting of the ..... 339
Amputation at Hip-Joint Necessitated by Caisson Fever. Twynam ..... 24
Amputation of the Thigh for Periosteal Sarcoma. Bell ..... 21
Anilin Poisoning ..... 232
Ano-Vesicle Centre ..... 192
Anthrarobin ..... 397
Antiseptics, Dangers of ..... 393
Antipyrine, Impure ..... 324
Australasia, Inter-Colonial Medical Congress of ..... 107, 109

### B

Baltimore Gynæcological and Obstetrical Society ..... 88, 151
Blennorrhagia, Gari Prize on ..... 264
Boards of Health, Conference of State ..... 366
Bogue, R. G. Two Cases of Abdominal Surgery ..... 4
Bologna, Italy, University's Eight Hundredth Anniversary ..... 325
Book Reviews ..... 60, 124, 196, 259, 313, 403
Books Received ..... 63, 127, 262, 407
Bow-Legs, Osteotomy for. Bell ..... 21
Brain Surgery ..... 193
Brazil, An English Sanitarium in ..... 320
British Medical Association, Research Scholarship of the ..... 323
British Medical Association, South Indian Branch of ..... 170
Broad Ligament, Primary Myoma of the. Bayard Holmes ..... 65
Broncho-Pneumonia. Johnson ..... 21
Brower. A Clinical Lecture ..... 273

### C

PAGE

Calculus Formed Around a Hair-pin in the Bladder of a Child ..... 23
Carbuncle, Excision and Scraping of ..... 306
Carcinoma of the Cervix. Fenger ..... 13
Carcinoma, Studies on the Etiology of ..... 285
Cardiac Failure in Pulmonary Obstruction. Thorowgood ..... 115
Cardiac Therapeutics, Present State of ..... 307
Charlatanism, Protest against Advertising of, by Religious Press ..... 402
Charter of College of Surgeons ..... 395
Chicago Gynæcological Society. Meeting of September 24, 1887 ..... 10
  Meeting of November 18, 1887 ..... 143
  Meeting of December 16, 1887 ..... 214
  Meeting of March 23, 1888 ..... 387
Chicago Medical College ..... 263
Chicago Medical Society. Meeting of November 7, 1887 ..... 99
  Meeting of January 3, 1888 ..... 376
  Meeting of April 2, 1888 ..... 379
  Meeting of April 16, 1888 ..... 380
Chicago Pathological Society. Meeting March 12, 1888 ..... 224
Cholera at New York ..... 120
Cholecystotomy; Recovery ..... 251
Chorea, Arsenic Subcutaneously in ..... 324
Circumcision in the Adult ..... 256
College of Physicians and Surgeons ..... 263
Color-Blindness a Brain Affection ..... 59
Condyloma of Vulva ..... 23
Congress of American Physicians and Surgeons ..... 264
Contamination by Sewage ..... 338
Cook County Directory of Physicians and Surgeons ..... 403
Cook County Hospital Internes, Alumni, Meeting of ..... 262
Cornea, Transplantation of ..... 311
Correspondence, Domestic. Licensing Medical Practitioners ..... 312
Correspondence, Foreign. Genoa ..... 171
Correspondence, Foreign. London Letter ..... 25
Crib and Tunnel under Lake Michigan at Chicago ..... 403
Curette, A new Uterine. Ashby ..... 95

D

PAGE

Dermatitis Papilaris, Dr. Hebra's Treatment 325
Diphtheria, Chloral in 392
Diphtheria, The Avine Origin of 398
Disinfection of Tuberculous Sputa 390
Diverting the Ureters and Removing the Bladder 247
Dogs, Danger of Keeping 59
Dyspnœa, Inhalation of Carbonic Acid Gas in 259

E

Eczema Simplex Dependent upon Ametropia. Richey 211
Edinburg Medico-Chirurgical Society 114, 168
Editiors, The Association of American Medical 365
Elevator, A New Uterine 214
Emperor William I, of Germany, Cause of Death of 325
Empyæma, Simple Puncture in 258
England, Right of Foreigners to Practice in 322
Epidemiology 257
Epithelial Growth With Colloid Degeneration. Jones 24
Erythrophlæine 393
Erythrophlæine as a Local Anæsthetic. Koller 201
Etheridge, J. H. Vaginal Hysterectomy 10

F

Faith Cures 400
Fenger, Christian. Carcinoma of the Cervix 13
Fenger, Christian. Cyst of the Pancreas 74
Filaria Sanguinis Hominis. Mackenzie 115
Fogs and Their Effects 182
Foreign Medical Matters 231
Frothingham, H. H. Three Cases of Nerve Injury; Operation, Recovery 209
Fundus, Dotted, in Night-Blindness. Nettleship 20

G

Gangrene of Finger from Pure Carbolic Acid 23
Garbage Crematory, Chicago's New 262
Garbage Crematory, The Chicago 324
Gastrotomy for Œsophageal Stricture. Chisholm 24
Genito-Unirary Surgeons, Preliminary Programme of Meeting of the American Association of 327
German Society of Naturalists and Physicians 111, 112, 200
Guy's Hospital Reports 183

H

Hæmaturia Simplex in a New-Born Child. Moyer 271

PAGE

Hæmorrhage into Anterior Chamber after Iridectomy. Emrys-Jones 19
Hair-Pin from the Urethra of a Girl 23
Havana, Small-Pox in 324
Head-Drop, A Curious Disease Called 116
Health Rules in Time of Cholera 253
Heart, Wound of the Left Ventricle; Recovery 308
Heathen Ignorance of the Healing Art 242
Hernia. Burchard 102
Hernia, Double Congenital Oblique Inguinal; MacEwan's Operation. Steele 276
Hernia, New Method of Reducing Strangulated 312
Hernia, Radical Cure of 58
Holmboe, A. Cyst of the Pancreas 74
Holmes, Bayard. Primary Myoma of the Broad Ligament 65
Holst, Leopold von 324
Horsewomen, A new Uterine Support for 123
Hospital Appointments in Chicago Hospitals 323
Hysterectomy, Vaginal. Etheridge 10, 100
Hysterectomy, Vaginal, for Sarcoma Uteri 216

I

Ileum, Tumor of 214
Illinois State Board of Health. Annual Meeting 78
Quarterly Meeting, April 19, 20, 1888 299
Illinois State Medical Society. Meeting of 1888 325
Thirty-eighth Annual Meeting of 367
Illinois State Medical Society, The 200
Injury, An Extraordinary 182
International Exhibition of Hygiene 200
International Otological Congress, The Fourth 200
Iodol, Value of 310
Inversion in Suspended Animation during Anæsthesia 245
Ireland, Royal Academy of Medicine in. Pathological Section 96, 169
Items 64, 128

J

Jaborandi as a Galactophore 123
Jenks, The Wm. F., Memorial Prize 198

K

"Kakke," Japanese 238
Keratitis, Stellate and Trabecular 122
Kidney, The Action of Drugs on the 54
Koller, Carl. Erythrophlæin as a Local Anæsthetic 201
Küstner, Professor, and Professor Wyder 324

L

PAGE

Lailler's Wash for Palpebral Eczema 324
Lano'in and its Uses 199
Laparotomy for Intestinal Obstruction; Recovery. Jones 24
Laparotomy, Report of Three Cases. Wilson 89
Larynx, Cystic Tumor of 309
Larynx, Extirpation of the 51
Lassar's Ointment for Scabies 324
Liver, Extirpation of Constricted Portion of 400
London, Harveian Society of 164
London, Medical Society of 115, 166
London, Obstetrical Soeiety of 162
London, Pathological Society of 160
London, Royal Medical and Chirurgical Society of 164

M

Malarial Germ of Lavaran. Councilman 105
Manchester, Clinical Society of 170
Medical Corps of the United States Navy 180
Medical Schools and Study in the United States 322
Menstruation from a Laparotomy Scar 311
Mercury, Alaninate of, a New Antisyphilitic 305
Michigan State Board of Health 177
Mistletoe, The American, as an Oxytocic and Emmenagogue. J. M. G. Carter 336
Montreal, Medico-Chirurgical Society of 21
Moyer, H. N. Hæmaturia Simplex in a New-Born Child 271
Murphy, J. B. Gunshot Wounds of the Abdomen 129
Mushroom Poisoning 310
Myoma, Primary, of the Broad Ligament 65

N

Naphthaline, The Action of, on the Visual Organs 195
Navy, Medical Corps of the 53
Navy, The Medical Corps of the United States 180
Nephrectomy. Parkes 100
Nephritis Secondary to Aortic Regurgitation. Robison 203
Nerve-Injury, Three Cases of; Operation, Recovery. Frothingham 209
New York Academy of Medicine 102
New South Wales, Medical Section of the Society of 23
Night-Blindness, Dotted Fundus in. Nettleship 20
Nymphomania, A Case of. Chunn 94

O

Obituary:
C. R. Agnew 325
E. G. Loring 325

PAGE

Ocular Movements, Congenital and Hereditary Defect of. Lawford 19
Onychia Parasitic. Bell 21
Operation for Abscess of the Spleen 249
Ophthalmia Neonatorum. Tilley 134
Ophthalmological Society of the United Kingdom 19
Orbit, Exostosis of. Silcock 20
Osteotomy for Bow-Legs. Bell 21

P

Pamphlets Received 127, 318, 407
Pancreas, Cyst of. Fenger and Holmboe 74
Pancreas, Cyst of the. Steele 205
Pennsylvania State Sanitary Convention 328
Pertussis. Robison 99
Phagedæna, Calomel in 310
Philadelphia, Pathological Society of 105
Phlegmasia Alba Dolens. Bennett 97
Phthisis, Antiseptic and Antipyretic Treatment of 254
Phthisis, On the Treatment by Creosote 303
Pilocarpine in Bright's Disease 258
Placenta Retained for Five Months, etc. Wing 334
Plumbing, Sanitary 323
Pocket-Case Antiseptic 259
Poisoning by the Ingestion of one Grain of Atropia Sulphate. Wescott 138
Poliomyelitis Anterior Acuta; Pseudo-Hypertrophic Paralysis; Sciatica—A Clinical Lecture. Brower 273
Polypus of the Tonsil 324
Prescription Writing, Greek Letter Delta in 325
Prize, The Gabrielle Bellion, of the "Académie des Sciences de Paris" 324
Ptomaines 41
Pupil as a Guide in the Administration of Chloroform 49
Pupil in Health and Disease 47

Q

Quarantine Does Not Deal With the Most Dangerous Diseases 181
Quarantine, National Control of Maritime 139
Quarantine, The New York 59

R

Rectum, Excision of the. Goode 24
Renal Stone and its Diagnosis 55
Report of the Supervising Surgeon-General of the United States Marine Hospital Service, 1887 36
Report of the Surgeon-General of the United States Army, 1887 28
Re-vaccination 321

PAGE
Reynolds, H. J. A New Method of Treating Vegetable Parasitic Diseases of the Skin 1
Richey, S. O. Eczema Simplex Dependent upon Ametropia 211
Robison, J.A. Nephritis Secondary to Aortic Regurgitation 203
Rome, Polyclinic in 324
Rotunda Hospital of Dublin, Report of 296
Royal College of Physicians, The Disciplinary Power of the 200
Rush Medical College Commencement 199

S

Saccharine 118
Saccharine "Tabloids" 119
Salicylate of Mercury in Venereal and Cutaneous Diseases 394
Salpingitis, Electrical Treatment in 257
Sanitary Convention at Manistee, Mich. 325
Sarcoma, Amputation of the Thigh for Periosteal. Bell 21
Scarlatina, Bacillus of 121
Septicæmia and Puerperal Thrombosis 311
Skin, A New Method of Treating Vegetable Parasitic Diseases of the. Reynolds 1
Society, Proposed Laryngological 199
Spartein, Sulphate of 255
Spirometer and Dynamometer 23
Spleen, Operation for Abscess of 249
Steele, D. A. K. A Clinical Lecture; Hernia 276
Steele, D. A. K. Cyst of the Pancreas 205
Stomach, Hair-Tumor of; Gastrotomy, Recovery 309
Stomach, Hour-glass Contraction of. Finney 96
Stomatitis, Treatment of Mercurial 401
Surgery, Two Cases of Abdominal. Bogue 4
Surgical Instruments, Import Duties on 198
Surgical Tuberculosis. Swartz 265
Swartz, T. B. Surgical Tuberculosis 265
Syphilis, Excision of Indurated Tissue and Enlarged Glands in 392
Syphilis, The Presence of Bacilli in 292
Syphilis, Unmerited 200

T

Tenotomy of External Rectus, Unusual Complication after 19
The Parkes Museum of Hygiene 400
Tibia, Marked Curvature of. MacCulloch 24
Tibia, Vicious Union of the. Waugh 24
Tilley, R. Ophthalmia Neonatorum 134
Trachea and Larynx, Subcutaneous Separation of 258
Training Schools for Attendants in Asylums for the Insane 263
Transfer of Nerve from Rabbit to Man 396

PAGE
Transmission of Tuberculosis from Human Subject to the Dog 391
Tumors, Etiology of. Smith 114

U

Uræmic Convulsions Treated by Pilocarpine 194
Uterine Elevator, A New 214
Uterus and Appendages, Removal of, for Uterine Fibroid 23
Uterus, Tetanoid Constriction of the. Wilson 88

V

Vaginal Hysterectomy. Etheridge 10
Visual Organs, The Action of Naphthaline on the 195

W

Water Gas, Protest against Introduction of, into Philadelphia 401
Water, Use of, at Meals 57
Weil's Disease 399
Wescott, C. D. Poisoning by the Ingestion of one Grain of Atropia Sulphate 138
Woman's Medical College 323
Word-Blindness 50
Wrinkles, A Cure for 122

### CONTRIBUTORS TO VOLUME LVI.

Bogue, R. G. Carter, J. M. G.
Fenger, Christian. Frothingham, H. H.
Holmes, Bayard. Holmboe, A.
Jackson, A. Reeves. Koller, Carl.
Moyer, H. N. Murphy, J. B.
Reynolds, H. J. Richey, S. O.
Robison, J. A. Swartz, T. B.
Steele, D. A. K. Tilley, R.
Wescott, C. D. Wing, Elbert.

Editorial:
Adulteration of Food 213
American Medical Association 337
Cyanosis Caused by Acetanilid 283
Dangers from Protracted Inhalation of Ether 282
Fraudulent Medical Advertising 142
Medical Appointments in Public Hospitals 7
Medical Legislation 7
Medical Progress in Italy 142
Medical Investigation and its Practical Results 6
National Control of Maritime Quarantine 139
Sanitary Matters in Michigan 214
Suits for Alleged Malpractice 283
The American Medical Association 141
The Amorphous Urate Deposit 212
The Index Medicus 213
The Multiplicity of Medical Societies 281
The Rush Monument Committee 284
The Surgeon-General of the Navy 284

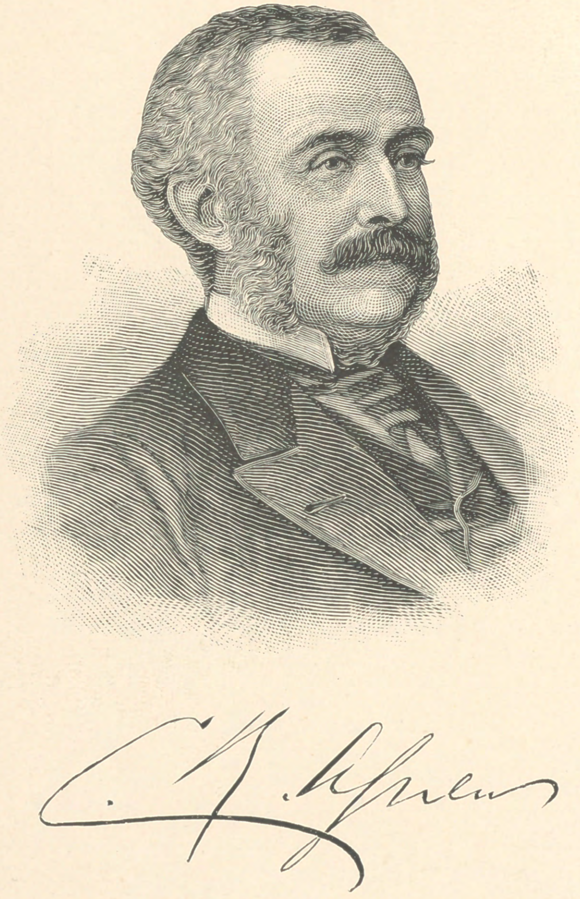

Professor C. R. Agnew, M. D.

### ANNOUNCEMENTS.

### CONGRESS OF AMERICAN PHYSICIANS AND SURGEONS.

### PRELIMINARY ANNOUNCEMENT OF THE COMMITTEE OF ARRANGEMENTS.

This association will hold its first triennial session in the city of Washington, during the 18th, 19th, and 20th of September next. The meetings of the Congress will be held in the evenings, beginning at 8 p. m., and those of the societies composing the Congress will be held during the daytime, according to the programme each may respectively provide. The sessions will be open to the profession.

The Local Committee of Arrangements of the Congress has secured places of meeting for the Congress and each society in close proximity, so that the members of the respective societies can interchange attendance at pleasure without inconvenience.

It is the purpose of the Executive Committee of the Congress to print the programmes of all of the societies, provided copies be supplied on or before Angust 15.

The Local Committee requests the secretaries of the societies to forward the names of those of their invited guests who have accepted their invitations, designating them as foreign and American.

The Committee of Arrangements is composed of one member of each society represented in the Congress, as follows:

Samuel C. Busey, Association of American Physicians, Washington.

J. Ford Thompson, American Surgical Association, Washington.

R. T. Edes, American Neurological Association, Washington.

E. C. Morgan, American Laryngological Association, Washington.

W. W. Johnston, American Climatological Association, Washington.

I. E. Atkinson, American Dermatological Association, Baltimore.

A. Sidney Roberts, American Orthopedic Association, Philadelphia.

H. Newell Martin, American Physiological Society, Baltimore.

Samuel Theobald, American Ophthalmological Society, Baltimore.

S. O. Richey, American Otological Society, Washington.

A. T. Cabot, American Association of Genito-Urinary Surgery, Boston.

Inquiries may be addressed to the Chairman, Dr. Busey, at Washington, or to the representative member of each society on the committee.

The Committee of Arrangements of the American Gynæcological Society, which will hold its next annual meeting in Washington at the same time, is composed of Drs. S. C. Busey, J. Taber Johnson, and A. F. A. King.

---

MEDICAL CONGRESS IN SPAIN. — The *Independencia Medica* states that a Medical Congress will be held at Barcelona from the 9th to the 15th of September, in connection with the Universal Exhibition which is shortly to be opened there. Among the subjects for discussion are the following: Measures to be taken by the state for the prevention and cure of blindness, and for the improvement of the condition of the blind in Spain; present state of leprosy in Spain, and how to prevent it from spreading; indications for surgical interference in intestinal obstruction; identity or difference of scrofula and tubercle; localization of lesions in diseases of nervous centres; application of hypnotism and "suggestion" to the treatment of nervous affections; micro-organisms in mineral waters, their influence on the chemical constitution, and therapeutic effects thereof; laparotomy as an exploratory measure in penetrating wounds of the abdomen (more especially gunshot wounds); antiseptic midwifery; etiology and prophylaxis of cholera and yellow fever. A Pharmaceutical Congress will meet at the same time.

---

An International Exposition of Hygiene and Life Saving and Preserving Apparatus will be opened in Ostend, Belgium, on June 3.

Volume LVI.] JUNE, 1888. [Number 6.

### The CHICAGO MEDICAL JOURNAL AND EXAMINER

EDITOR:
S. J. JONES, M.D., LL.D

COLLABORATORS:

CHICAGO:

Professor E. ANDREWS, M.D., LL.D.
Professor J. BARTLETT, M.D.
Professor W. T. BELFIELD, M.D.
Professor F. BILLINGS, M.D.
Professor W. H. BYFORD, A.M., M.D.
Professor L. CURTIS, A.M., M.D.
Professor N. S. DAVIS, Jr., A.M., M.D.
Professor O. C. DeWOLF, A.M., M.D.
Professor E. C. DUDLEY, A.B., M.D.
Professor C. FENGER, M.D.
Professor J. N. HYDE, A.M., M.D.
Professor E. F. INGALS, A.M., M.D.
Professor F. S. JOHNSON, A.M., M.D.
Professor H. A. JOHNSON, M.D., LL.D.
Professor C. W. PURDY, M.D.
Professor C. G. SMITH, A.M., M.D.
Professor E. WING, A.M., M.D

MILWAUKEE, WIS.:

Professor N. SENN, M.D., Ph.D.

WASHINGTON, D. C.:
S. O. RICHEY, M.D.

UNITED STATES ARMY:
SURGEON D. L. HUNTINGTON, M.D.

UNITED STATES NAVY:
SURGEON-GENERAL J. M. BROWNE, M.D.
MEDICAL-DIRECTOR A. L. GIHON, A.M., M.D.

MARINE HOSPITAL SERVICE:
SURGEON S. T. ARMSTRONG, M.D., Ph.D.

